# A simulation‐based method for evaluating geometric tests of a linac c‐arm in quality control in radiotherapy

**DOI:** 10.1002/acm2.12698

**Published:** 2019-09-14

**Authors:** Mateusz Baran, Krzysztof Rzecki, Damian Kabat, Monika Tulik, Anna Wydra, Zuzanna Derda, Agata Sochaczewska, Zbisław Tabor

**Affiliations:** ^1^ Cracow University of Technology Kraków Poland; ^2^ Center of Oncology Maria Sklodowska‐Curie Memorial Institute Kraków Branch Poland; ^3^ OptiNav Sp. z o.o. Słupsk Poland

**Keywords:** geometric tests, isocenter, linac, quality assurance, radiotherapy

## Abstract

**Purpose:**

Assessment of the accuracy of geometric tests of a linac used in external beam therapy is crucial for ensuring precise dose delivery. In this paper, a new simulation‐based method for assessing accuracy of such geometric tests is proposed and evaluated on a set of testing procedures.

**Methods:**

Linac geometry testing methods used in this study are based on an established design of a two‐module phantom. Electronic portal imaging device (EPID) images of fiducial balls contained in these modules can be used to automatically reconstruct linac geometry. The projection of the phantom modules fiducial balls onto the EPID detector plane is simulated for assumed nominal geometry of a linac. Then, random errors are added to the coordinates of the projections of the centers of the fiducial balls and the linac geometry is reconstructed from these data.

**Results:**

Reconstruction is performed for a set of geometric test designs and it is shown how the dispersion of the reconstructed values of geometric parameters depends on the design of a geometric test. Assuming realistic accuracy of EPID image analysis, it is shown that for selected testing plans the reconstruction accuracy of geometric parameters can be significantly better than commonly used action thresholds for these parameters.

**Conclusions:**

Proposed solution has the potential to improve geometric testing design and practice. It is an important part of a fully automated geometric testing solution.

## 1 Introduction

A precise dose delivery to a target location during radiotherapy is a crucial requirement of an external beam therapy. This requirement may be met provided that the geometry of the beam agrees with the geometry defined in the treatment planning system (TPS).

In practice, the nominal geometry of a linac as defined in TPS agrees with the real geometry of the device only to a limited accuracy. The discrepancies between a nominal and a real geometry have the source in the construction of linear accelerator components and especially in their enormous weights leading to flexing or sagging of the system components during gantry rotation.^1^ To keep dose delivery errors at a sufficiently low level, the discrepancies between a nominal and a real geometry must not exceed strict tolerance limits. To check whether the tolerance limits are met, a detailed inspection of the operation of each of the movable elements of a linac is periodically conducted as a part of quality assurance programs in radiotherapy departments.^2^

Each country has its own legislation or recommendations for the quality tests, for example, in a form of papers prepared by national societies of medical physicists which specify types of tests to perform, their frequency, and proposed tolerance values, based on international guidelines.^2^, ^3^, ^4^ The most important geometric tests include estimation of the position of isocenter, jaws offsets, and precision of movements of a gantry, a collimator and a couch.

A generic procedure for a geometric test (e.g., the Winston‐Lutz test) consists of positioning of a special phantom containing one or more radiopaque fiducial markers in a radiation beam, acquisition of MeV images of the phantom (most frequently electronic portal imaging device (EPID) images) and then—based on dedicated image analysis methods—determination of the quantities of interest like isocenter position, source to detector distance (SDD) etc.^1^, ^5^, ^6^, ^7^, ^8^, ^9^, ^10^, ^11^, ^12^, ^13^ Finally, the value of the geometric parameter as computed from the test results (e.g., EPID images) is compared against the assumed nominal value. If the difference exceeds an action threshold, the device does not pass the test.

There are at least three weak points in this procedure. First, the EPID images offer limited resolution with a pixel size of only about 0.35 mm. The geometric tests are based on prior determination of the positions of the projection of the centers of phantom fiducials onto the EPID image plane. Consequently, the accuracy of the consecutive analysis steps and in particular accuracy of the estimation of geometric parameters are limited by the resolution of EPID detectors. Next, the EPID images are corrupted by noise and for this reason analysis of two projection images of a phantom acquired under exactly the same geometric settings may return different results of geometric testing. Finally, there is no systematic way for setting action thresholds for the values of the geometric parameters. In particular, without knowing how big the random errors introduced by an image noise and systematic errors introduced by limited EPID resolution are, it is not clear if the accuracy requirements can be met or, contrary, if the requirements are too loose posing a hazard of a malfunctioning device passing a test.

This paper is an extension of a previous work^13^ where a design of a multimodular phantom is presented and a set of reconstruction procedures for this phantom is briefly analyzed. In this paper, we focus on the problem of how the design of a geometric testing procedure and phantom influences the accuracy of estimation of parameters related to the geometry of linac C‐arms. We show in simulations how the distributions of the results of geometric tests depend on testing plans and uncertainties related to EPID imaging. This is, to the best of our knowledge, the first work considering the influence of inaccuracies of imaging on the accuracy of geometric tests.^5^, ^6^ Based on simulation results, we postulate a probability‐based extension to the geometric tests of linear accelerators. In particular, given an actual value of a difference between a nominal and a measured value of a geometric parameter (for example the isocenter position or the size of a radiation field), one may estimate some probability associated with this value for a device with all subsystems correctly set and for uncertainty of the geometric parameter measurement introduced only at the stage of imaging of a phantom used in geometrical testing (as explained above, this uncertainty may arise for example due to noise and finite resolution of EPID used to capture phantom images). From this probability, one may infer if the device operates correctly or if there are other factors besides uncertainties in image acquisition (e.g., a malfunctioning subsystem of a linac) which account for the observed discrepancies between a nominal and an observed value of a geometric parameter under test. In the present paper, we consider geometry testing procedures based on a phantom proposed in an earlier paper,^13^ however the proposed approach can be applied to other phantoms described in the literature as well.

## 2 Materials and methods

### 2.1 Simulations

In this work, the process of projecting fiducial markers onto the EPID matrix is simulated, taking into account errors caused by finite resolution of the detector and noise inherently associated with imaging process. In the simulations, we assume that all the subsystems of a simulated device are set correctly, that is, there is no difference between nominal and actual device settings like gantry or collimator angle, SDD, jaw positions, and other. Acquisition of images of a multimodule multifiducial phantom^13^ by an EPID is simulated using these nominal settings. The phantom^13^ consists of two modules, the first one, mounted on a treatment table, contains a set *B *= {*B_k_*: *k* = 1, 2, …, *N_B_*} of *N_B_* fiducial balls and the second one, mounted on a gantry head, contains a set *C* = {*C_l_*: *l* = 1, 2,..., *N_C_*} of *N_C_* fiducial balls.^13^

Let *Q* denote a parameter which must be estimated during a geometrical testing of a linac (e.g., isocenter position). *Q*, like any other geometrical parameter, has some preset nominal value *Q_N_*, which is used to simulate the process of acquisition of phantom EPID images. We denote by *Q_M_* a value of *Q* estimated based on analysis of EPID images.

Due to inherent noise present in images captured by ionizing radiation detectors, *Q_M_* is a random variable sampled from some distribution with probability density function *f_Q_*(x|*P_T_*, *B*, *C*, σ) where *P_T_* describes the testing procedure and σ is the standard deviation quantifying the uncertainty of the EPID image analysis. The procedure *P_T_* = {(*θ_i_*, *ψ_j_*): *i* = 1, 2,..., *N_Θ_*, *j* = 1, 2,..., *Nψ*} of geometrical testing of a linac consists of a set of pairs of collimator angles *θ_i_* and gantry angles *ψ_j_*. The testing procedure depends also on other geometrical settings like table position or rotation. These settings were fixed in the simulations, although both table translation and rotation can be determined from the projection images of the phantom used in the simulation.^13^

The nominal coordinates of the projections of the centers of *B_k_* and *C_l_* onto the EPID imaging plane are determined for each element of *P_T_* using nominal settings of a linac. It is assumed that the measured coordinates *P*(*B_k_*) and *P*(*C_l_*) of the projections of centers of *B_k_* and *C_l_*, respectively, onto the EPID imaging plane are random variables with a two‐dimensional symmetric Gaussian distribution centered at the nominal projection points of the centers of the ball markers and standard deviation equal to σ.

Given the measured coordinates *P*(*B_k_*) and *P*(*C_l_*) of the projections of the centers of fiducial markers *B_k_* and *C_l_* for each element of *P_T_*, *Q* is recomputed from these data resulting in *Q_M_*. *Q_M_* differs from *Q_N_* to an extent that depends on how big the uncertainty σ is, on the phantom design (i.e., on *B* and *C*) and on the testing procedure *P_T_*. While *P_T_*, *B* and *C* are a part of the designed testing procedure, the value of σ can be reduced by for example increasing the irradiation time or by increasing the resolution of the EPID detector matrix.

Fixing *P_T_*, *B*, *C,* and σ and repeating the simulation multiple times, we find the probability density function *f_Q_*(*x*|*P_T_*, *B*, *C*, σ) of the measured values. *Q_M_* of *Q*. *f_Q_*(*x*|*P_T_*, *B*, *C*, σ) is then used to determine how the selection of testing procedure *P_T_*, the phantom design *B* and *C* and the uncertainty of image analysis σ influence the accuracy of estimating *Q*.

Further details concerning the simulations and reconstruction of the geometry of the linac are described in Data S1.

### 2.2 Geometrical quantities

Following quantities are computed based on simulation results:

1. Radiation isocenter position. To find the radiation isocenter *I*, rotation axes of the collimator are determined for a sequence *ψ* = {*ψ*_1_, *ψ*_2_,..., *ψ_N_*} of angular positions of a gantry. Let these axes be denoted by *R*_1_, *R*_2_,..., *R_N_*. Then, the position of the isocenter is defined as follows:
  (1) $$I={\text{argmin}}_{P\in R^{3}}\sum_{i=1}^{N}d{\left(P,R_{i}\right)}^{2},$$
  where *d*(*P*, *R*_*i*_) is the distance from a point P to the axis *R*_*i*_. Nominally, isocenter is located at the origin, that is, the point with coordinates (0, 0, 0). From the simulations, we determine the distribution of the distance from the nominal to the calculated position of the isocenter as well as distributions of the components of the calculated isocenter position. The reconstruction procedure is performed using Algorithm 2 from Data S1.
2. Source to detector distance, which is the distance from the source of the MeV radiation to the EPID detector plane. The nominal value of *SDD* is 180 cm. The value of SDD is calculated based on the isocenter position calculated using Algorithm 2 and equation of the detector plane obtained using Algorithm 4 from Data S1.
3. Source to axis distance (*SAD*), which is the distance from the source to the projection of the source onto the plane normal to the rotation axis of the collimator and containing the isocenter. The nominal value of *SAD* is 100 cm. It is calculated using isocenter position, reconstructed collimator axis and reconstructed radiation source position (see Algorithms 2 and 4).
4. Gantry rotation axis. Nominally, gantry rotation axis is equal to a vector (0, 1, 0), that is a vector parallel to the Y axis of the coordinate frame of the isocenter. First, positions of fiducial ball markers and radiation sources are collected for a set of gantry angles and collimator angles. Gantry rotation axis is estimated by calculating the extrinsic mean of directions of optimal rotations of collected points for different gantry angles (see Algorithm 2 in Data S1).
5. Gantry rotation angle, which is the angle between the local vertical direction and the axis of rotation of the collimator for the actual gantry angular position (see Algorithms 1 and 4 in Data S1).
6. The radiation field size. To find the radiation field size for some fixed angular position of the gantry, one determines the rotation axis *R* of the collimator, the source position *S* and the coordinates of the projections *A_P_*, *B_P_*, *C_P_*, and *D_P_* of the corners of the radiation field onto the detector plane. Then, one finds a plane *H*, normal to *R* and containing the isocenter *I*. Then for each line *A_P_S*, *B_P_S*, *C_P_S*, and *D_P_S* one finds its intersection with *H*. The projection points are the corners *A*, *B*, *C*, and *D* of the radiation field in the isocentric plane *H*. From the simulations, we determine the distances between the nominal *A_N_*, *B_N_*, *C_N_*, and *D_N_* and calculated positions of the corners *A*, *B*, *C*, and *D* of the radiation field, the distribution of the lengths of the edges *AB*, *BC*, *CD*, *DA* of the radiation field and the distribution of the angles *ABC*, *BCD*, *CDA*, *DAB* formed by the consecutive edges of the radiation field. In the simulations, we fix the radiation field size to a square 20 cm × 20 cm.

## 3 Results

The simulations were run for experimental plans consisting of from 5 to 25 gantry angles (in the range from −180° to 180°, an interval from 15° to 90°), from 5 to 25 collimator angles (in the range from −165° to 165°, an interval from 15° to 90°). Random selection of gantry and collimator angles was also tested. The number of fiducial balls of phantom modules was varied from 4 to 8 for the collimator‐mounted module and from 6 to 10 for the table‐mounted module. The fiducial markers of the table‐mounted module were organized in parallel planes, at most four markers within one plane. All used markers had the radius of 3 mm. In any case it was checked that all the markers are projected onto EPID for every angular position of the gantry head contained in the testing plan and that their projections onto EPID do not overlap (to fulfill the last condition it is enough that each marker has a different coordinate along the rotation axis of the gantry). In the case of a collimator‐mounted module, the ball markers are projected onto EPID provided that they are within the radiation field. The fiducial markers of the collimator‐mounted module were also organized in parallel planes, at most three markers within one plane. The standard deviation of the uncertainty of determination of the projection of the phantom balls centers was varied in the range from 0.1 to 0.5 mm. While the actual size of an EPID pixel depends on an EPID model, currently, the smallest size of an EPID pixel is approximately equal to 0.35 mm.

**Figure 1:**
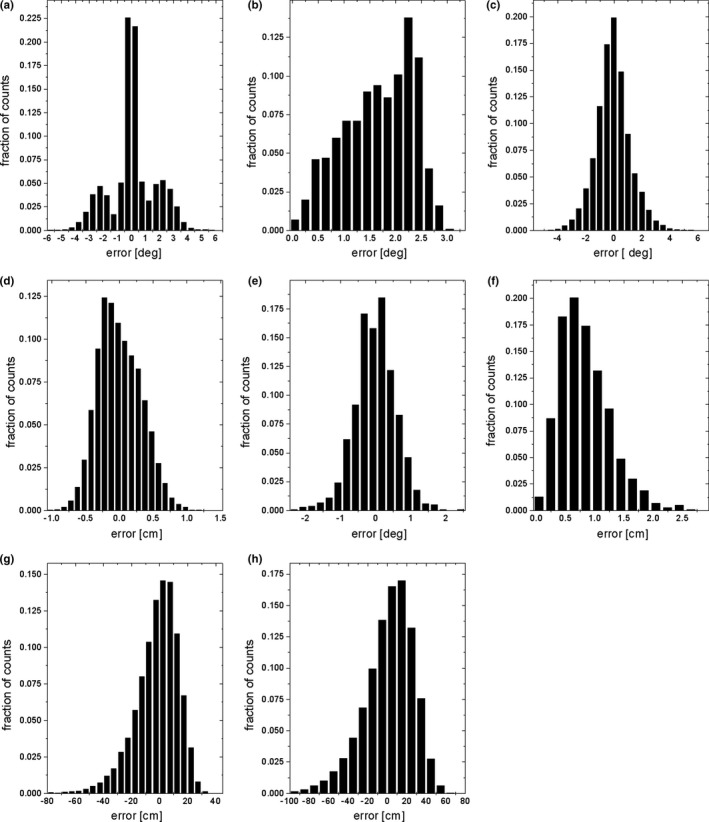
Histograms of estimation errors of controlled geometrical quantities: differences *α* between estimated and nominal angle between the rotation axis of the collimator and a vertical direction (a), angles *θ* between estimated and nominal axes of gantry rotation (b), differences *ω* between estimated and nominal angles formed by the edges of a radiation field in an isocentric plane (c), differences between estimated and nominal lengths *L* of the edges of a radiation field in an isocentric plane (d), estimated components *s* of the position of the isocenter in space (e), estimated distances *l* of the isocenter from the nominal position (f), differences between estimated and nominal *SAD *(g), differences between estimated and nominal *SDD *(h).

In Fig. 1 histograms of the estimation errors of geometrical quantities with respect to the nominal values of these quantities are plotted. The plots demonstrate the results found for five collimator angles (−165°, −90°, 0°, 90°, and 165°), 25 gantry angles (in the range from −180° to 180° with the interval equal to 15°), the standard deviation of the uncertainty of determination of the projection of the phantom balls centers equals to 0.5 mm, six balls in the table‐mounted phantom module and four balls in the collimator‐mounted phantom module. The balls of the table‐mounted module were enclosed within a cube of size 8 cm × 8 cm × 8 cm. The balls of the collimator‐mounted module were enclosed within a cube of size 6 cm × 6 cm × 6 cm.

In Tables 1, 2, 3, 4, 5, 6, 7, we show how the standard deviations of the errors of estimation of geometrical quantities with respect to the nominal values of these quantities depend on the number of gantry and collimator angles used to estimate these quantities and on the uncertainty in estimating the coordinates of the projection of the phantom fiducial balls onto the detector plane. With the number of measurements exceeding one thousand, the confidence limits for the standard deviations are approximately ±5%. The values of the standard deviations do not depend on the actual selection of the collimator or gantry angles: random selection of these angles in the accessible range leads to similar results provided that the number of selected angles remains fixed. The values of the standard deviations do not depend also on the actual placement of the markers as long as the volume occupied by the markers remains fixed and the markers are projected onto EPID for all gantry angles used in the analysis. The results presented in Tables 1, 2, 3, 4, 5, 6, 7 were calculated for six balls in a table‐mounted phantom module and four balls in a collimator‐mounted phantom module. The balls of the table‐mounted module were enclosed within a cube of size 8 cm × 8 cm × 8 cm. The balls of the collimator‐mounted module were enclosed within a cube of size 6 cm × 6 cm × 6 cm. In Tables 8 and 9 we show how the standard deviation of the differences between the estimated and nominal values of the geometrical parameters depends on the number of balls in a collimator and in a table‐mounted modules, respectively. These results were found for nine gantry angles and 25 collimator angles. The balls of the table‐mounted module were enclosed within a cube of size 8 cm × 8 cm × 8 cm. The balls of the collimator‐mounted module were enclosed within a cube size of size 6 cm × 6 cm × 6 cm.

**Table 1:** Standard deviations (in degrees) of *α*, that is, the difference between estimated and nominal angle between the rotation axis of the collimator and a vertical direction.

Number of gantry angles	Number of collimator angles	σ = 0.1 mm	σ = 0.2 mm	σ = 0.3 mm	σ = 0.4 mm	σ = 0.5 mm
25	5	0.083	0.236	1.290	5.420	13.20
25	9	0.060	0.138	0.409	1.160	5.290
25	25	0.037	0.083	0.227	0.461	0.765
9	5	0.088	0.226	0.960	6.470	15.10
9	9	0.064	0.139	0.447	1.080	3.590
9	25	0.040	0.088	0.238	0.500	0.822
5	5	0.093	0.200	0.875	4.000	14.60
5	9	0.069	0.151	0.456	1.060	6.660
5	25	0.042	0.090	0.240	0.506	0.853

**Table 2:** Standard deviations of angles *θ* between estimated and nominal rotation axis of a gantry.

Number of gantry angles	Number of collimator angles	σ = 0.1 mm	σ = 0.2 mm	σ = 0.3 mm	σ = 0.4 mm	σ = 0.5 mm
25	5	0.66	0.67	0.64	0.64	0.67
25	9	0.69	0.69	0.69	0.71	0.71
25	25	0.72	0.71	0.72	0.73	0.76
9	5	2.16	2.16	2.10	2.12	2.17
9	9	2.19	2.28	2.22	2.29	2.33
9	25	2.31	2.25	2.34	2.34	2.41
5	5	4.81	4.84	4.87	4.73	4.94
5	9	5.10	5.02	4.91	5.12	5.11
5	25	5.21	5.13	5.03	5.41	5.26

**Table 3:** Standard deviations (in degrees) of *ω*, that is, the difference between estimated and nominal angle formed by the edges of a radiation field in an isocentric plane.

Number of gantry angles	Number of collimator angles	σ = 0.1 mm	σ = 0.2 mm	σ = 0.3 mm	σ = 0.4 mm	σ = 0.5 mm
25	5	0.29	0.52	0.75	0.97	1.21
25	9	0.29	0.53	0.75	0.99	1.21
25	25	0.29	0.52	0.75	0.98	1.22
9	5	0.47	0.65	0.83	1.04	1.26
9	9	0.46	0.63	0.84	1.04	1.24
9	25	0.45	0.63	0.83	1.03	1.24
5	5	0.86	0.96	1.09	1.25	1.44
5	9	0.83	0.94	1.07	1.22	1.42
5	25	0.82	0.91	1.07	1.21	1.40

**Table 4:** Standard deviations (in centimeters) of the differences *L* between estimated and nominal lengths of the edges of a radiation field in an isocentric plane.

Number of gantry angles	Number of collimator angles	σ = 0.1 mm	σ = 0.2 mm	σ = 0.3 mm	σ = 0.4 mm	σ = 0.5 mm
25	5	0.073	0.130	0.189	0.252	0.323
25	9	0.072	0.130	0.187	0.248	0.309
25	25	0.072	0.129	0.187	0.245	0.304
9	5	0.124	0.166	0.218	0.287	0.374
9	9	0.122	0.162	0.213	0.269	0.332
9	25	0.118	0.161	0.212	0.262	0.317
5	5	0.301	0.320	0.349	0.416	0.500
5	9	0.298	0.315	0.346	0.388	0.444
5	25	0.304	0.313	0.348	0.381	0.423

**Table 5:** Standard deviations (in centimeters) of the estimated components *s* of the position of the isocenter in space.

Number of gantry angles	Number of collimator angles	σ = 0.1 mm	σ = 0.2 mm	σ = 0.3 mm	σ = 0.4 mm	σ = 0.5 mm
25	5	0.012	0.034	0.125	0.311	0.537
25	9	0.009	0.021	0.063	0.152	0.313
25	25	0.005	0.012	0.034	0.072	0.124
9	5	0.020	0.053	0.238	0.518	0.890
9	9	0.015	0.034	0.108	0.254	0.506
9	25	0.009	0.021	0.058	0.119	0.201
5	5	0.027	0.062	0.262	0.714	1.163
5	9	0.020	0.043	0.137	0.330	0.604
5	25	0.012	0.027	0.073	0.153	0.260

**Table 6:** Standard deviations (in centimeters) of the differences between estimated and nominal *SAD.*

Number of gantry angles	Number of collimator angles	σ = 0.1 mm	σ = 0.2 mm	σ = 0.3 mm	σ = 0.4 mm	σ = 0.5 mm
25	5	2.7	5.7	8.7	11.8	15.7
25	9	2.7	5.7	8.7	11.8	15.4
25	25	2.7	5.7	8.6	11.9	15.5
9	5	2.8	5.6	8.5	12.1	15.5
9	9	2.8	5.6	8.7	11.9	15.3
9	25	2.8	5.7	8.8	11.8	15.7
5	5	2.7	5.5	8.1	11.4	14.2
5	9	2.8	5.4	8.3	11.2	14.6
5	25	2.7	5.4	8.2	11.2	14.8

**Table 7:** Standard deviations (in centimeters) of the differences between estimated and nominal *SDD.*

Number of gantry angles	Number of collimator angles	σ = 0.1 mm	σ = 0.2 mm	σ = 0.3 mm	σ = 0.4 mm	σ = 0.5 mm
25	5	4.9	10.1	15.3	20.5	26.2
25	9	4.9	10.1	15.4	20.5	25.9
25	25	4.9	10.1	15.1	20.6	26.0
9	5	5.0	10.0	15.0	21.0	26.1
9	9	5.0	10.0	15.4	20.6	25.8
9	25	5.0	10.1	15.5	20.4	26.3
5	5	4.9	9.9	14.4	19.7	24.3
5	9	5.0	9.8	14.8	19.6	24.4
5	25	4.9	9.8	14.5	19.6	25.2

**Table 8:** Standard deviations (in degrees for parameters in rows 1 to 3 and in centimeters for parameters in rows 4 to 7) of the differences between estimated and nominal values of geometrical parameter in function of the number of balls in the collimator‐mounted phantom module.

Parameter	Number of balls in the collimator module	σ = 0.1 mm	σ = 0.2 mm	σ = 0.3 mm	σ = 0.4 mm	σ = 0.5 mm
α	4	0.040	0.088	0.238	0.500	0.822
	6	0.021	0.041	0.065	0.097	0.134
	8	0.016	0.031	0.052	0.085	0.137
θ	4	2.31	2.25	2.34	2.34	2.41
	6	2.37	2.39	2.36	2.36	2.41
	8	2.31	2.34	2.35	2.30	2.41
ω	4	0.45	0.63	0.83	1.03	1.24
	6	0.45	0.62	0.83	1.04	1.25
	8	0.45	0.62	0.83	1.04	1.25
L	4	0.12	0.16	0.21	0.26	0.32
	6	0.12	0.16	0.21	0.26	0.32
	8	0.12	0.16	0.21	0.26	0.31
s	4	0.009	0.021	0.058	0.119	0.201
	6	0.004	0.008	0.014	0.023	0.032
	8	0.002	0.005	0.009	0.017	0.031
SAD	4	2.8	5.7	8.8	11.8	15.7
	6	2.8	5.6	8.6	11.8	15.2
	8	2.7	5.6	8.7	11.9	15.5
SDD	4	5.0	10.1	15.5	20.4	26.3
	6	5.0	9.9	15.3	20.4	25.7
	8	4.9	9.9	15.3	20.7	26.1

**Table 9:** Standard deviations (in degrees for parameters in rows 1 to 3 and in centimeters for parameters in rows 4 to 7) of the differences between estimated and nominal values of geometrical parameter in function of the number of balls in a table‐mounted phantom module.

Parameter	Number of balls in the table module	σ = 0.1 mm	σ = 0.2 mm	σ = 0.3 mm	σ = 0.4 mm	σ = 0.5 mm
*α*	6	0.040	0.088	0.238	0.500	0.822
	8	0.034	0.070	0.154	0.329	0.568
	10	0.032	0.064	0.130	0.282	0.482
*θ*	6	2.3	2.3	2.3	2.3	2.4
	8	2.3	2.3	2.3	2.4	2.4
	10	2.3	2.3	2.3	2.3	2.4
*ω*	6	0.45	0.63	0.83	1.03	1.24
	8	0.42	0.57	0.74	0.91	1.11
	10	0.42	0.55	0.70	0.87	1.04
*L*	6	0.12	0.16	0.21	0.26	0.32
	8	0.11	0.15	0.19	0.23	0.28
	10	0.11	0.14	0.18	0.22	0.26
*s*	6	0.009	0.021	0.058	0.119	0.201
	8	0.008	0.016	0.034	0.075	0.129
	10	0.007	0.014	0.029	0.062	0.108
*SAD*	6	2.8	5.7	8.8	11.8	15.7
	8	2.3	4.6	7.0	9.5	12.2
	10	2.1	4.3	6.4	8.5	10.9
*SDD*	6	5.0	10.1	15.5	20.4	26.3
	8	4.1	8.3	12.5	16.8	21.2
	10	3.7	7.6	11.4	15.0	18.9

**Figure 2:**
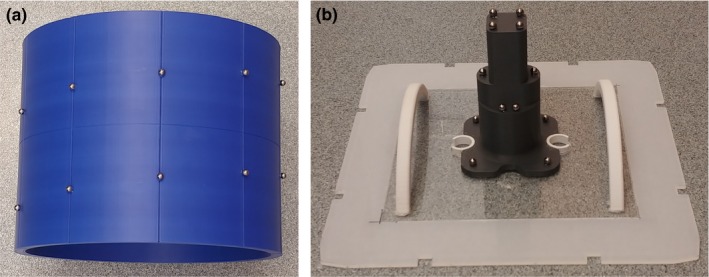
Configuration of balls in the two phantom modules: the table‐mounted phantom module (a) and the collimator‐mounted phantom module (b).

In the final experiment, we considered a case of a bigger phantom, occupying a cuboid of size 26 cm × 18 cm × 26 cm, containing 18 fiducial balls in the table‐mounted module and eight fiducial balls in the collimator‐mounted module. The balls of the table‐mounted module were distributed along a spiral pattern whose axis was parallel to the axis of a gantry rotation. The fiducial markers of the collimator‐mounted module were contained in parallel planes, at most four markers within each plane. The configuration of both modules is depicted in Fig. 2. Smaller modules considered in other experiments had similar configuration. To ensure that all the fiducial markers of the table‐mounted module are projected onto the EPID plane, *SDD* was decreased from 180 cm as in previous numerical experiments to 140 cm. The simulations were run for gantry and collimator angles incremented by 5°. In Table 10, we show the values of standard deviation of the differences between the estimated and nominal values of the geometrical parameters calculated for such testing plan.

**Table 10:** Standard deviations (in degrees for parameters in rows 1 to 3 and in centimeters for parameters in rows 4 to 7) of the differences between estimated and nominal values of geometrical parameter for a big phantom modules.

Parameter	σ = 0.1 mm	σ = 0.2 mm	σ = 0.3 mm	σ = 0.4 mm	σ = 0.5 mm
α	0.004	0.009	0.014	0.019	0.023
θ	0.01	0.11	0.26	0.32	0.31
ω	0.05	0.12	0.18	0.24	0.30
L	0.02	0.039	0.068	0.095	0.12
s	0.0004	0.0009	0.0014	0.0017	0.0022
SAD	0.25	0.51	0.76	1.01	1.28
SDD	0.32	0.71	1.08	1.43	1.80

## 4 Discussion

In this paper, we demonstrated how the dispersion of geometric parameters derived from the projection images of a specialized phantom depends on the design of a testing procedure and the design of the phantom. The simulations were run assuming that there is an error in estimating the coordinates of the projections of phantom markers onto the EPID detector plane. This error was modeled as a Gaussian random variable with standard deviation ranging from 0.1 to 0.5 mm. The smallest size of pixel in currently used EPID devices is approximately equal to 0.35 mm. Given the state‐of‐the‐art image analysis methods like circle Hough transform and knowing the approximate position of each marker already from the nominal settings of a linac, it can be safely agreed that the error in determining the coordinates of the projections of phantom markers onto an EPID detector plane will be never greater than ±2 pixels. For reference, Du and Yang^14^ developed a method for center localization based on Hough Transform with accuracy below 1 pixel even for small phantom markers and noisy images. Also, another method^15^ can be used to locate ellipses with mean Hausdorff distance of 2.3 pixels to ground truth, which would result in even more accurate determination of ellipse centers. As for Gaussian distributions almost 100% of results fall within ± 3 σ range, it can be reasonably assumed that the results found for σ = 0.2 mm and σ = 0.3 mm correspond to clinical settings.

The construction of phantom modules together with the procedure used in these simulations guarantee that the procedure is not influenced by certain types of error that have to be taken into account in standard geometry tests.^13^ For example, the procedure does not assume accurate positioning of the phantom modules while errors in determining the positions of fiducial markers can be made negligibly small by the means of precise measurement of their real positions using coordinate‐measurement devices. Moreover, certain systematic errors can be detected by the procedure, for example the sagging during gantry rotation. The precise determination of the source of an error that can be detected is not our goal and therefore such errors do not need to be explicitly modeled.

It is important to assess how the design of the testing procedure and the design of the phantom influence the accuracy of a measurement of geometrical parameters. The accuracy of a measurement of the angle between the axis of rotation of a collimator and a local vertical direction increases with increasing the number of collimator angles included in the testing procedure, the number of markers within the phantom modules and with phantom size. By increasing the number of collimator angles from 9 to 25, the error *α* is reduced by approximately 40%. By increasing the number of markers within the collimator‐mounted module by a factor of two, the error *α* is reduced by a factor of three. These dependencies are clear as the axis of collimator rotation is determined from the coordinates of the markers of the collimator‐mounted module. To determine these coordinates, the projections of the markers of the table‐mounted module are used. For this reason, the accuracy of the measurement of the angle between the axis of rotation of a collimator and a local vertical direction must depend on the design of the table‐mounted module, in particular on its size and the number of markers. For almost minimal set of markers and a bigger phantom modules, it is possible to reduce the error *α* to approximately 0.02 degrees for clinical settings and 25 collimator angles used in a testing procedure.

The accuracy of a measurement of the deviation *θ* between nominal and calculated axis of gantry rotation depends on the number of gantry angles used to estimate it. With 25 gantry angles error *θ* is approximately equal to 0.7°. Increasing number of gantry angles by a factor of two decreases error *θ* by the same factor. With such a trend it follows that probing gantry angles at an interval of 1 degree, the error of estimation of the gantry rotation axis corresponds to about 1.5 mm deviation at 2 m distance from the isocenter.

The value of the angular difference *ω* between the estimated and nominal angle formed by the edges of a radiation field in an isocentric plane depends weakly on the number of gantry angles and the number of balls within the table‐mounted module. It is an indirect dependence as the contours of a radiation field are determined for a plane containing the isocenter and the accuracy in determining isocenter depends on the number of gantry angles. The measurement error *ω* depends more strongly on phantom size. With bigger phantom modules the error of a measurement *ω* can be reduced to 0.2° that correspond to 0.35 mm (one pixel) for a 10 cm × 10 cm radiation field.

The values of differences *L* between estimated and nominal lengths of the edges of a radiation field in an isocentric plane depend on the same components of the testing procedure as *ω*. With the bigger phantom design, the error of a measurement *L* can be reduced to 0.5 mm which is slightly larger than an EPID pixel.

The values of the components *s* of the isocenter position in space depend on all components of the testing plan and on the phantom design. Increasing the number of gantry and collimator angles, the number of ball markers and the phantom size decreases the error *s* to different extents. It, however, follows that even for not very complex testing plans it is possible to reduce the error *s* to a submillimeter range.

The pattern of the dependence of the measurement error of *SAD* and *SDD* on the testing plan and the phantom design is the same. The errors of both *SAD* and *SDD* are independent of the number of collimator and gantry angles and on the number of balls in a collimator‐mounted module. This follows directly from the method of applying the phantom for estimating the geometrical parameters^13^. These errors are weakly reduced by increasing the number of balls of a table‐mounted phantom module and strongly reduced by increasing the size of a table‐mounted phantom module. The size of a phantom is however limited by the maximal size of a radiation field and by the geometrical constraints. For a single measurement using the big phantom and decreased nominal *SDD,* the error of *SAD* is of the order of 0.8 cm. Better estimation of *SAD* or *SDD* requires averaging over multiple measurements instead of using the result of a single measurement. If one averages values of *SAD* over, for example, 100 collimator angles (these kind of data must be collected anyway to find the rotation axis of a collimator), then the error of estimation of an averaged *SAD* decreases to submillimeter accuracy.

It follows from the presented results that even when the resolution of an EPID is limited, it is still possible to perform geometric testing with satisfactory accuracy by proper selection of an architecture of the phantom and a testing plan. The presented results may provide guidelines for selection of a testing plan to achieve the submillimeter and subdegree accuracy of geometric testing procedures used in quality control in radiotherapy.

### 4.1 Future work

The simulation tools developed in the present study and the results presented can be used in a future study to design a probabilistic framework for testing the geometry of a linac in a following way. The geometry testing usually involves comparison of *Q_M_* to the nominal value *Q_N_* of a parameter *Q*. For this reason one is, in practice, interested in how big the absolute value of the difference between *Q_M_* and *Q_N_* is. Thus, to evaluate the results of a geometric test we have to calculate the probability *Pr* (|*Q* – *Q_N_*| = Δ ≥ Δ*_M_* = |*Q_M_* – *Q_N_|*):

(2) $$\mathrm{Pr}\left(\Delta \geq \Delta_{M}\right)=\int_{-\infty }^{2Q_{N}-Q_{M}}f_{Q}\left(x\left|P_{T},B,C,\sigma \right.\right)dx+\int_{Q_{M}}^{\infty }f_{Q}\left(x\left|P_{T},B,C,\sigma \right.\right)dx,$$

where Pr(Δ ≥ Δ*_M_*) is the probability that in a device operating according to the assumed model (defined by nominal settings) the observed difference between measured value of *Q* and a *Q_N_* is greater than Δ*_M_*. Clearly, if Pr(Δ ≥ Δ*_M_*) becomes too low, one may strongly conclude that the deviation of an actual device from the model is too serious and appropriate measures must be taken to correct it.

Then, to apply a geometry testing procedure, one may select a probability threshold Pr_TH_ and if Pr(Δ ≥ Δ*_M_*) is less than Pr_TH_, then one concludes that the geometrical test has failed because for the used testing plan P_T_ the probability of observing an actual difference Δ*_M_* in a correctly tuned device is lower than an accepted threshold Pr_TH_. Equivalently, one may define a tolerated difference Δ_TH_ by:

(3) $$\mathrm{Pr}{}_{\text{TH}}=\int_{-\infty }^{Q_{N}-\Delta_{\text{TH}}}f_{Q}\left(x\left|P_{T},B,C,\sigma \right.\right)\text{d}x+\int_{Q_{N}+\Delta_{\text{TH}}}^{\infty }f_{Q}\left(x\left|P_{T},B,C,\sigma \right.\right)\text{d}x,$$

and conclude that the test has failed if Δ*_M_* is larger than Δ*_TH_*. The distribution functions and probabilities can be derived directly from the simulation results, as demonstrated in the previous section.

Within the probabilistic framework described above questions about a testing plan, *P_T_* can be stated and answered in a future study. First, assume that the value of a tolerated difference Δ_TH_ must be respected due to some factors of clinical relevance. If a testing plan *P_T_* is not properly designed, the measured differences Δ*_M_* can be larger than Δ_TH_ in substantial percentage of cases of correctly tuned devices due to purely random factors. Thus one may ask what must be the testing plan *P_t,T_* if it is required that the assumed value of Δ_TH_ corresponds to some user‐specified probability threshold Pr_TH_. In other words, both Pr_TH_ and Δ_TH_ are given and only procedures *P_t,T_* such that

(4) $$\mathrm{Pr}{}_{\text{TH}}\geq \int_{-\infty }^{Q_{N}-\Delta_{\text{TH}}}f_{Q}\left(x\left|P_{t,T},B,C,\sigma \right.\right)\text{d}x+\int_{Q_{N}+\Delta_{\text{TH}}}^{\infty }f_{Q}\left(x\left|P_{t,T}B,C,\sigma \right.\right)\text{d}x,$$

are admissible. In practice, a testing procedure will be selected from the set of admissible plans for geometrical testing guided by some requirement, for example minimal number of gantry and collimator angles necessary to achieve the expected goal.

The final problem that may be also addressed in future within a probabilistic testing framework is the independence of measurements of various parameters. In particular, assume that two geometrical parameters, say *Q*_1_ and *Q*_2_ are estimated resulting in measured values *Q*_1_ *_,M_* and *Q*_2_ *_,M_*, while the nominal values for *Q*_1_ and *Q*_2_ are *Q*_1_ *_,N_* and *Q*_2_ *_,N_*. We are interested in how the discrepancies Δ*_1,M_* = *|Q*_1_ *_,M_*–*Q*_1_ *_,N_|* and Δ_2,_ *_M_* = *|Q*_2_ *_,M_*–*Q*_2_ *_,N_|* are correlated. In particular, for measurement discrepancies that are not independent it is possible that observing some specific pair of *Q*_1_ *_,M_* and *Q*_2_ *_,M_* is an unlikely event but such conclusion cannot be drawn from the analysis of the distributions of *Q*_1_ and *Q*_2_ separately. In this analysis, the random variable *QJ* = (*Q*_1_, *Q*_2_) with joint probability density function $f_{\mathrm{QJ}}\left(x,y|P_{T},B,C,\sigma \right)$ must be considered.

In the context of geometrical testing, another important problem can be formulated. Assume that the decision that a device passes or fails some geometrical test involving measurement of some geometrical parameter is made based on a preselected threshold probability Pr_TH_, the geometrical testing procedure may be complemented by a requirement that if an expected value of the geometric parameter differs from the nominal value by more than some acceptance threshold Δ_TH_, then such an event must be detected with a probability Pow_TH_. Now, if the testing procedure must meet constraints about both Pr_TH_ and Pow_TH_, the problem is how to select an appropriate testing plan *P_T_* to address these requirements. While the answer to this question is certainly important, it would require simulation of possible mechanical failures of a linac, for example to estimate the distribution of a geometrical quantity of interest in both correctly tuned and malfunctioning devices (e.g., a device which introduces a random error with an expected value of Δ_TH_ to the measured quantity). Our simulation does not address such cases because modeling failures of a linac are not possible without detailed knowledge of its mechanical design.

## 5 Conclusions

In this paper, a new simulation‐based method for assessing accuracy of geometric tests of a linac is proposed and evaluated on a set of testing procedures. Parameters describing geometry of a linac are reconstructed from the projections of the centers of fiducial balls of a two‐module phantom after adding random errors to the coordinates of these projections. Assuming realistic accuracy of EPID image analysis it is shown that for selected testing plans the reconstruction accuracy of geometric parameters can be significantly better than commonly used action thresholds for these parameters. Proposed solution has the potential to improve geometric testing design.

## Conflict of interest

The authors have no relevant conflicts of interest to disclose.

## Supporting information


**Data S1.** Reconstruction procedures.
