# Supplementary material for: A simulation‐based method for evaluating geometric tests of a linac c‐arm in quality control in radiotherapy
Source: J Appl Clin Med Phys. 2019 Sep 14;20(9):133–42. doi: 10.1002/acm2.12698 (PMC6753736; doi:10.1002/acm2.12698)
Supplement: Supplementary file 1 — Data S1. Reconstruction procedures. [file ACM2-20-133-s001.pdf]

Supplementary material for the paper ”A simulation-based method for evaluating geometric tests of a linac c-arm in quality control in radiotherapy”

(Dated: July 17, 2019)

I. RECONSTRUCTION PROCEDURES

Following are general procedures of reconstruction of geometric parameters of a linac. These procedures assume a more general test plan $P_T = (U, \Theta, \Phi, \Psi_C, \Psi_T, T_X, T_Y, T_Z)$ composed of default device settings U, a set $\Theta = \{\theta_i : i = 1, 2, \ldots, N_\Theta\}$ of collimator angles, a set $\Phi = \{\phi_j : j = 1, 2, \ldots, N_\Phi\}$ of couch angles, two sets Ψ_C, Ψ_T of gantry angles and three sets T_X, T_Y, T_Z of couch translations. The device settings $U = (\psi_U, \theta_U, \phi_U, t_X, t_Y, t_Z, c_{X,1}, c_{X,2}, c_{Y,1}, c_{Y,2}, t_{EPIDMeV,z})$ describe the configuration of a linac, namely the gantry angle ψ_U, collimator angle θ_U, couch angle ϕ_U, couch translation (t_X, t_Y, t_Z), collimator jaw positions $c_{X,1}$, $c_{X,2}$, $c_{Y,1}$ and $c_{Y,2}$ and position of the MeV EPID $t_{EPID,z}$. For tests described in the paper sets Φ, Ψ_T, T_X, T_Y and T_z are not used and U is the same for all tests.

The main algorithm, defining the order in which other subprocedures are performed, is described by Alg. 1. Specific steps are performed by subsequent procedures. Reconstruction of isocenter and the Y axis is done using Alg. 2. This is followed by calculation of the Z axis using Alg. 8. Next, positions of jaws of the collimator are calculated by Alg. 11. Finally, reconstruction of translations of the couch is performed by Alg. 12.

Algorithm 1: General procedure of reconstruction of geometric parameters of a linac.

Data: Test plan P_T.

Result: Reconstructed geometric parameters.

reconstruct isocenter O and the Y axis v_Y;

reconstruct the Z axis v_Z;

`/* Reconstruction of the X axis by cross product. */`

$v_X = v_Z \times v_Y$;

reconstruct positions of corners of the radiation field $J_{C,all}$;

reconstruct selected translations $T_{X,obs}$, $T_{Y,obs}$, $T_{Z,obs}$ of the couch;

return O, v_X, v_Y, v_Z, $J_{C,all}$, $T_{X,obs}$, $T_{Y,obs}$, $T_{Z,obs}$

Reconstruction for device settings U described by Alg. 4 determines the position Q_S of the source of the MeV radiation, basis vectors λ_x, λ_y of the imaging plane and its normal vector λ_z, origin Q_I of the imaging plane, constant C_{EPID} of the imaging plane equation $\lambda_z \cdot (x, y, z) = C_{EPID}$, coordinates $\hat{C} = \{\hat{C}_l : l = 1, 2, \ldots, N_C\}$ of the collimator mounted

Algorithm 2: Reconstruction of the isocenter O and the Y axis v_Y.

Data: Test plan $P_T = (U, \Theta, \Phi, \Psi_C, \Psi_T, T_X, T_Y, T_Z)$.

Result: Isocenter O and the Y axis v_Y.

set collimator rotation axis set CRS to an empty set;

set collimator rotation center set CRC to an empty set;

set gantry rotation set GRS to an empty set;

set reference gantry rotation point set $RGRC$ to an empty set;

for $i \leftarrow 1$ **to** N_{Ψ_C} **do**

  $U' \leftarrow U$;

  change gantry angle in U' to $\psi_{C,i}$;

  `/* Rotation parameters are calculated using Alg. 3. */`

  $MRS, MRC, CGRC \leftarrow$ calculate rotation parameters for U';

  `/* Mean axis algorithm uses the extrinsic mean on the unit sphere. */`

  $v_{mean} \leftarrow$ mean of axes from MRS;

  $c_{mean} \leftarrow$ mean of points from MRC;

  add v_{mean} to CRS;

  add c_{mean} to CRC;

  if $i = 1$ **then**

    $RGRC \leftarrow CGRC$;

  else

    $(v_{M,x}, v_{M,y}, v_{M,z}), w_{opt}, t_{opt}, \alpha \leftarrow$ result of optimal the rotation algorithm for sequences $RGRC$ and $CGRC$;

    add $((v_{M,x}, v_{M,y}, v_{M,z}), w_{opt})$ to GRS;

  end

end

/* Summation performed over all lines L given by CRS and CRC. $d(\hat{O}, L)$ denotes the Euclidean distance between point $\hat{O}$ and line L. */

$O \leftarrow \arg\min_{\hat{O} \in \mathbb{R}^3} \sum_L d(\hat{O}, L)^2$;

$v_Y \leftarrow$ extrinsic mean of directions of lines from GRS, represented as points in $\mathbb{R}^3$;

return O, v_Y

Algorithm 3: Reconstruction of rotation parameters.

Data: Test plan $P_T = (U, \Theta, \Phi, \Psi_C, \Psi_T, T_X, T_Y, T_Z)$, device settings U'

Result: Rotation parameters.

set rotation axis set MRS to an empty set;
set rotation center set MRC to an empty set;
set current gantry rotation point set $CGRC$ to an empty set;
for $j \leftarrow 1$ **to** N_Θ **do**
    $U'' \leftarrow U'$;
    set collimator angle in U'' to θ_j;
    $Q_S, \hat{C}, Q_{M2}, v_{M,z} \leftarrow$ reconstruct geometry for U'';
    add $v_{M,z}$ to MRS;
    add Q_{M2} to MRC;
    if $j = 1$ **then**
        add Q_S to $CGRC$
    end
    add all points from $\hat{C}$ to $CGRC$;
end
return $MRS, MRC, CGRC$

phantom module balls, center Q_{M2} of the collimator mounted phantom module and its basis vectors $v_{M,x}$, $v_{M,y}$, $v_{M,z}$. All coordinates are given in the reference frame of the table mounted phantom module.

The optimal rotation computation performed by Alg. 7 was implemented using the SVD decomposition approach [1]. It uses the Eigen library [2] to compute the SVD decomposition.

---

1. Lorusso A, Eggert DW, Fisher RB. A Comparison of Four Algorithms for Estimating 3-D Rigid Transformations. In: Proceedings of the 1995 British Conference on Machine Vision (Vol. 1). BMVC '95. Surrey, UK, UK: BMVA Press; 1995:237–246. http://dl.acm.org/citation.cfm?id=236190.236213. Accessed September 11, 2018.
2. Guennebaud G, Jacob B, et al. Eigen v3. http://eigen.tuxfamily.org, 2010.

Algorithm 4: Reconstruction of geometry from ball projections on the EPID matrix for device settings U.

Data: Device settings U.

Result: Values of Q_S, λ_x, λ_y, λ_z, Q_I, C_{EPID}, $\hat{C}$, Q_{M2}, $v_{M,x}$, $v_{M,y}$ and $v_{M,z}$.

measure coordinates $\{P_{B,k} : k = 1, 2, \ldots, N_B\}$ and $\{P_{C,l} : l = 1, 2, \ldots, N_C\}$ of ball projections on the imaging plane;

/* x_1 contains coordinates of the source of the MeV radiation, spherical coordinates of λ_x and λ_y and a constant C where $\lambda_z \cdot (x, y, z) = C$ is the equation of the imaging plane. All parameters are computed for a correctly fuctioning device for settings U. */

$x_1 \leftarrow (Q_{S,x}, Q_{S,y}, Q_{S,z}, \lambda_{x,\theta}, \lambda_{x,\phi}, \lambda_{y,\theta}, \lambda_{y,\phi}, C_{EPID})$;

/* Reconstruct values of Q_S, λ_x, λ_y and C_{EPID} for actually measured positions of projections of ball centers. */

$x_{opt} \leftarrow$ result of optimization of the objective function obj_1 initialized by x_1 with ball coordinates $\{B_k : k = 1, 2, \ldots, N_B\}$ and measured ball projections $\{P_{B,k} : k = 1, 2, \ldots, N_B\}$;

extract Q_S, λ_x, λ_y and C_{EPID} from x_{opt};

$\lambda_z = \lambda_x \times \lambda_y$;

$Q_I \leftarrow P_{B,1} + \frac{C_{EPID} - P_{B,1} \cdot \lambda_z}{P_{B,1} \cdot \lambda_z - Q_S \cdot \lambda_z}(P_{B,1} - Q_S)$;

/* τ_1 contains numbers $\tau_{1,l}$ for $l = 1, 2, \ldots, N_C$ such that for each lth ball of the collimator mounted phantom module $Q_S + \tau_l(C_l - Q_S)$ belongs to the imaging plane. */

$\tau_1 \leftarrow (\tau_{1,l} : l = 1, 2, \ldots, N_C)$;

$\tau_{opt} \leftarrow$ result of optimization of the objective function obj_2 initialized by τ_1 with projections $\{P_{C,l} : l = 1, 2, \ldots, N_C\}$ of ball centers, MeV source position Q_S, origin of EPID MeV Q_I and EPID MeV basis vectors λ_x, λ_y;

$\hat{C} \leftarrow (Q_S + \tau_{opt,l}(Q_I + P_{C,l,x}\lambda_x + P_{C,l,y}\lambda_y - Q_S))_{l=1}^{N_C}$;

$(v_{M,x}, v_{M,y}, v_{M,z}), w_{opt}, t_{opt}, \alpha \leftarrow$ result of optimal the rotation algorithm for sequences $(C_i)_{i=1}^{N_C}$ and $\hat{C}$;

return Q_S, λ_x, λ_y, λ_z, Q_I, C_{EPID}, $\hat{C}$, t_{opt}, $v_{M,x}$, $v_{M,y}$, $v_{M,z}$

Algorithm 5: Calculation of value of the obj_1 objective function for input x, ball coordinates $\{B_k : l = 1, 2, \ldots, N_B\}$ and measured projections of ball centers $\{P_{B,k} : k = 1, 2, \ldots, N_B\}$.

Data: Optimization parameters $(Q_{S,x}, Q_{S,y}, Q_{S,z}, \lambda_{x,\theta}, \lambda_{x,\phi}, \lambda_{y,\theta}, \lambda_{y,\phi}, C_{EPID})$, ball centers $\{B_k : k = 1, 2, \ldots, N_B\}$ and measured projections of ball centers $\{P_{B,k} : k = 1, 2, \ldots, N_B\}$.

Result: Value of the objective function obj_1.

`/* Conversion from spherical coordinates to cartesian coordinates. */`

$E_x \leftarrow \lambda_x$;

$E_y \leftarrow \lambda_y$;

`/* Vector normal to the imaging plane. */`

$E_z \leftarrow E_x \times E_y$;

$o \leftarrow 0$;

for $i \leftarrow 2$ **to** N_B **do**

  `/* Predicted distance. */`

  $\Delta \leftarrow B_i + \frac{C_{EPID} - E_z \cdot B_i}{E_z \cdot B_i - E_z \cdot Q_S}(B_i - Q_S) - B_1 - \frac{C_{EPID} - E_z \cdot B_1}{E_z \cdot B_1 - E_z \cdot Q_S}(B_1 - Q_S)$;

  `/* Measured distance. */`

  $\Delta_M \leftarrow E_x(P_{B,i,x} - P_{B,1,x}) + E_y(P_{B,i,y} - P_{B,1,y})$;

  `/* Add squared Euclidean norm of the difference. */`

  $o \leftarrow o + \|\Delta - \Delta_M\|^2$;

end

return o

Algorithm 6: Calculation of value of the obj_2 objective function for input τ_1, ball coordinates $\{C_l : l = 1, 2, \ldots, N_C\}$ and measured projections of ball centers $\{P_{C,l} : l = 1, 2, \ldots, N_C\}$, MeV source position Q_S, origin of the EPID MeV Q_I and EPID MeV basis vectors λ_x, λ_y.

Data: Optimization parameters $\tau_1 = (\tau_{1,l} : l = 1, 2, \ldots, N_C)$, projections of ball centers $\{P_{C,l} : l = 1, 2, \ldots, N_C\}$, MeV source position Q_S, origin of the EPID MeV Q_I and EPID MeV basis vectors λ_x, λ_y.

Result: Value of the objective function obj_2.

$o \leftarrow 0$;
for $i \leftarrow 1$ **to** N_C **do**
  for $j \leftarrow 1$ **to** N_C **do**
    if $i \neq j$ **then**
      $R_i \leftarrow Q_S + \tau_{1,i}(Q_I + P_{C,i,x}\lambda_x + P_{C,i,y}\lambda_y - Q_S)$;
      $R_j \leftarrow Q_S + \tau_{1,j}(Q_I + P_{C,j,x}\lambda_x + P_{C,j,y}\lambda_y - Q_S)$;
      `/* Predicted squared distance. */`
      $\Delta \leftarrow \|C_i - C_j\|^2$;
      `/* Measured squared distance. */`
      $\Delta_M \leftarrow \|R_i - R_j\|^2$;
      `/* Add squared difference. */`
      $o \leftarrow o + (\Delta - \Delta_M)^2$;
    end
  end
end
return o

Algorithm 7: Optimal rotation algorithm.

Data: Two sequences of vectors $(u_i)_{i=1}^M$, $(v_i)_{i=1}^M$.

Result: Standard Euclidean basis rotated by the optimal rotation, rotation axis, rotation angle and optimal translation.

$(R_{opt}, t_{opt}) \leftarrow \arg\min_{R \in SO(3), t \in \mathbb{R}^3} \sum_{i=1}^M \|Ru_i + t - v_i\|^2$;

$w_{opt} \leftarrow$ rotation axis of R_{opt};

$\alpha \leftarrow$ rotation angle of R_{opt};

return $(R_{opt}(1,0,0), R_{opt}(0,1,0), R_{opt}(0,0,1))$, w_{opt}, t_{opt}, α;

Algorithm 8: Reconstruction of the Z axis.

Data: Test plan $P_T = (U, \Theta, \Phi, \Psi_C, \Psi_T, T_X, T_Y, T_Z)$.

Result: Coordinates of the Z axis vector v_Z in the reference frame of the table mounted phantom module.

set table rotation set TRS to an empty set;
set reference gantry point set RGP to an empty set;
for $i \leftarrow 1$ **to** N_Φ **do**
  $U' \leftarrow U$;
  change table angle in U' to ϕ_i;
  `/* Reconstruction performed by Alg. 9. */`
  $CGP \leftarrow$ reconstruct gantry point set for device settings U';
  if $i = 1$ **then**
    $GRP \leftarrow CRP$;
  else
    `/* Selection using Alg. 10. */`
    $RSEL, CSEL \leftarrow$ select current and reference gantry point sets from RGP and CGP;
    $(v_{M,x}, v_{M,y}, v_{M,z}), w_{opt}, t_{opt}, \alpha \leftarrow$ result of optimal the rotation algorithm for sequences $RSEL$ and $CSEL$;
    `/* Rotations by very small angles do not provide relevant rotation axes. This happens when gantry rotations` -180 `and 180 degrees are simultaneously in` Ψ_T`. */`
    if $\alpha > 1 \times 10^{-8}$ rad **then**
      add w_{opt} to TRS;
    end
  end
end
$v_Z \leftarrow$ extrinsic mean of directions of vectors from TRS, represented as points in $\mathbb{R}^3$;
return v_Z

Algorithm 9: Reconstruction of the current gantry point set.

Data: Test plan $P_T = (U, \Theta, \Phi, \Psi_C, \Psi_T, T_X, T_Y, T_Z)$, device settings U'.

Result: Gantry point set for U'.

set current gantry point set CGP to a sequence of N_{Ψ_T} empty sets;
for $j \leftarrow 1$ **to** N_{Ψ_T} **do**
    $U'' \leftarrow U'$;
    set gantry angle in U'' to $\psi_{T,j}$;
    if *settings* U'' *are physically achievable* **then**
        $Q_S, \hat{C}, Q_{M2}, v_{M,z} \leftarrow$ reconstruct geometry for U'';
        add all points from $\hat{C}$ and Q_S to jth element CGP;
    end
end
return CGP

Algorithm 10: Selection of current and reference points.

Data: Test plan $P_T = (U, \Theta, \Phi, \Psi_C, \Psi_T, T_X, T_Y, T_Z)$, reference gantry point set RGP, current gantry point set CGP.

Result: Selected current and reference points.

set the selected reference point set $RSEL$ to an empty set;
set the selected current point set $CSEL$ to an empty set;
for $j \leftarrow 1$ **to** N_{Ψ_T} **do**
    if j*th elements of* RGP *and* CGP *are not empty* **then**
        add points from jth element of RGP to $RSEL$;
        add points from jth element of CGP to $CSEL$;
    end
end
return $RSEL$, $CSEL$

Algorithm 11: Jaw position reconstruction.

Data: Test plan $P_T = (U, \Theta, \Phi, \Psi_C, \Psi_T, T_X, T_Y, T_Z)$, isocenter O, position of MeV radiation source O_S, axes v_X and v_Y.

Result: Positions of corners of the radiation field on the plane spanned by v_X and v_Y through the isocenter O.

set $J_{C,all}$ to an empty list;
for *corner* J_C *of the jaw of collimator* **do**
    measure position P_{J_C} of the projection of J_C on the EPID MeV;
    $\hat{J}_C \leftarrow O_I + P_{J_C,x}\lambda_x + P_{J_C,y}\lambda_y$;
    $\hat{J}_{C,P} \leftarrow$ common point of plane $\{O_I + t_x v_X + t_y v_Y : t_x, t_y \in \mathbb{R}\}$ and line connecting O_S and $\hat{J}_C$;
    add $\hat{J}_{C,P}$ to $J_{C,all}$;
end
return $J_{C,all}$

Algorithm 12: Couch translation reconstruction.

Data: Test plan $P_T = (U, \Theta, \Phi, \Psi_C, \Psi_T, T_X, T_Y, T_Z)$.

Result: Real translations $T_{X,obs}$, $T_{Y,obs}$, $T_{Z,obs}$ measured for the given plan.

set $T_{X,obs}$, $T_{Y,obs}$, $T_{Z,obs}$ to empty lists;
for *axis* $A \in$ *(X, Y, Z)* **do**
    $U' \leftarrow U$;
    set gantry angle of U' to 0.0;
    $Q_{M2,ref} \leftarrow$ reconstruct geometry for U';
    for $i = 1$ **to** N_{T_A} **do**
        set couch position of U' along axis A to $T_{A,i}$;
        $Q_{M2} \leftarrow$ reconstruct geometry for U';
        add $Q_{M2,ref} - Q_{M2}$ to $T_{A,obs}$;
    end
end
return $T_{X,obs}$, $T_{Y,obs}$, $T_{Z,obs}$
